# Early anti-TNF/immunomodulator therapy is associated with better long-term clinical outcomes in Asian patients with Crohn’s disease with poor prognostic factors

**DOI:** 10.1371/journal.pone.0177479

**Published:** 2017-05-23

**Authors:** Eun Hye Oh, Kyunghwan Oh, Minkyu Han, Hyungil Seo, Kiju Chang, Sun-Ho Lee, Gwang-Un Kim, Eun Mi Song, Myeongsook Seo, Ho-Su Lee, Sung Wook Hwang, Sang Hyoung Park, Dong-Hoon Yang, Kyung-Jo Kim, Jeong-Sik Byeon, Seung-Jae Myung, Suk-Kyun Yang, Byong Duk Ye

**Affiliations:** 1 Department of Internal Medicine, University of Ulsan College of Medicine, Asan Medical Center, Seoul, Korea; 2 Department of Clinical Epidemiology and Biostatistics, University of Ulsan College of Medicine, Seoul, Korea; 3 Department of Gastroenterology, University of Ulsan College of Medicine, Asan Medical Center, Seoul, Korea; 4 Health Screening and Promotion Center, University of Ulsan College of Medicine, Asan Medical Center, Seoul, Korea; 5 Inflammatory Bowel Disease Center, University of Ulsan College of Medicine, Asan Medical Center, Seoul, Korea; University Hospital Llandough, UNITED KINGDOM

## Abstract

Although early treatment of Crohn’s disease (CD) patients with anti-tumor necrosis factor (TNF) agents or immunomodulators (IMs) may improve long-term outcomes, especially those with poor prognostic factors, their effectiveness in Asians remains unclear. In this study, Korean patients with CD naïve to both intestinal surgery and intestinal complications, and with at least two risk factors for progression (diagnosis at age <40 years, systemic corticosteroid treatment <3 months after diagnosis, and perianal fistula at diagnosis) were retrospectively analyzed. Patients were classified into those who started anti-TNFs, or IMs but not anti-TNFs, within 2 years of diagnosis, and those who started anti-TNFs and/or IMs later. Their probabilities of intestinal surgery and intestinal complications were compared. A total of 670 patients were enrolled, 79 in the early anti-TNF, 286 in the early IM, and 305 in the late treatment group. Kaplan-Meier analysis with the log-rank test showed that from starting anti-TNFs/IMs, times to intestinal surgery (*P* < 0.001), stricturing complications (*P* = 0.002), and penetrating complications (*P* < 0.001) were significantly longer in the early anti-TNF/IM groups than in the late treatment group. Multivariate Cox regression analysis showed that, from starting anti-TNFs/IMs, late anti-TNF/IM treatment was independently associated with higher risks of intestinal surgery (adjusted hazard ratio [aHR] 2.321, 95% confidence interval [CI] 1.503–3.584, *P* < 0.001), behavioral progression (aHR 2.001, 95% CI 1.449–2.763, *P* < 0.001), stricturing complications (aHR 1.736, 95% CI 1.209–2.493, *P* = 0.003), and penetrating complications (aHR 3.315, 95% CI 2.094–5.249, *P* < 0.001) than early treatment. In conclusion, treatment of Asian CD patients having poor prognostic factors with anti-TNFs/IMs within 2 years of diagnosis is associated with better clinical outcomes than later treatment.

## Introduction

Crohn’s disease (CD) is a chronic systemic inflammatory disease that mainly affects the gastrointestinal tract, although it also has multiple extra-intestinal manifestations [1]. CD is relatively common in Western countries, with a prevalence of up to 0.5% in the general population [2]. Although it is not as common in Asian countries, its incidence and prevalence in East Asian countries have increased markedly over the past decades and are expected to increase more in the future [3, 4].

The introduction of anti-tumor necrosis factor (TNF) agents has greatly changed the treatment paradigm of patients with inflammatory bowel disease (IBD), including CD. These agents are effective in inducing and maintaining clinical remission of active IBD [5–9]. Mucosal healing induced by these drugs is associated with better clinical outcomes [10, 11]. Moreover, the effectiveness of anti-TNF agents may be greater if they are used early in the course of disease, and in combination with immunomodulators (IMs) [12–14].

Despite these benefits of anti-TNF drugs, they are not indicated for all patients with CD, due to their adverse effects and costs [15, 16]. It is therefore necessary to select patients who would benefit from these more potent agents, both in controlling disease activity and preventing disease progression [17, 18]. Several factors are associated with poor prognosis in patients with CD, including young age at diagnosis, perianal disease at diagnosis, upper gastrointestinal tract involvement, early need for systemic corticosteroids, and smoking [19, 20]. These factors can be used to select patients requiring treatment with anti-TNFs, even if these patients show mild disease activity at diagnosis. The concept of “top-down therapy” has been proposed, in which effective biologics are started at the first attack of disease in patients with poor prognostic factors [21].

Most studies on therapeutic strategies in treating CD have been performed in Western countries. Applying these strategies to Asian patients with CD requires more evidence about the effectiveness of the early use of anti-TNF agents and IMs in Asian cohorts. This study therefore retrospectively analyzed the effects of early anti-TNF or IM therapy on long-term outcomes in Korean patients with CD and poor prognostic factors.

## Materials and methods

### Study population

**Fig 1:**
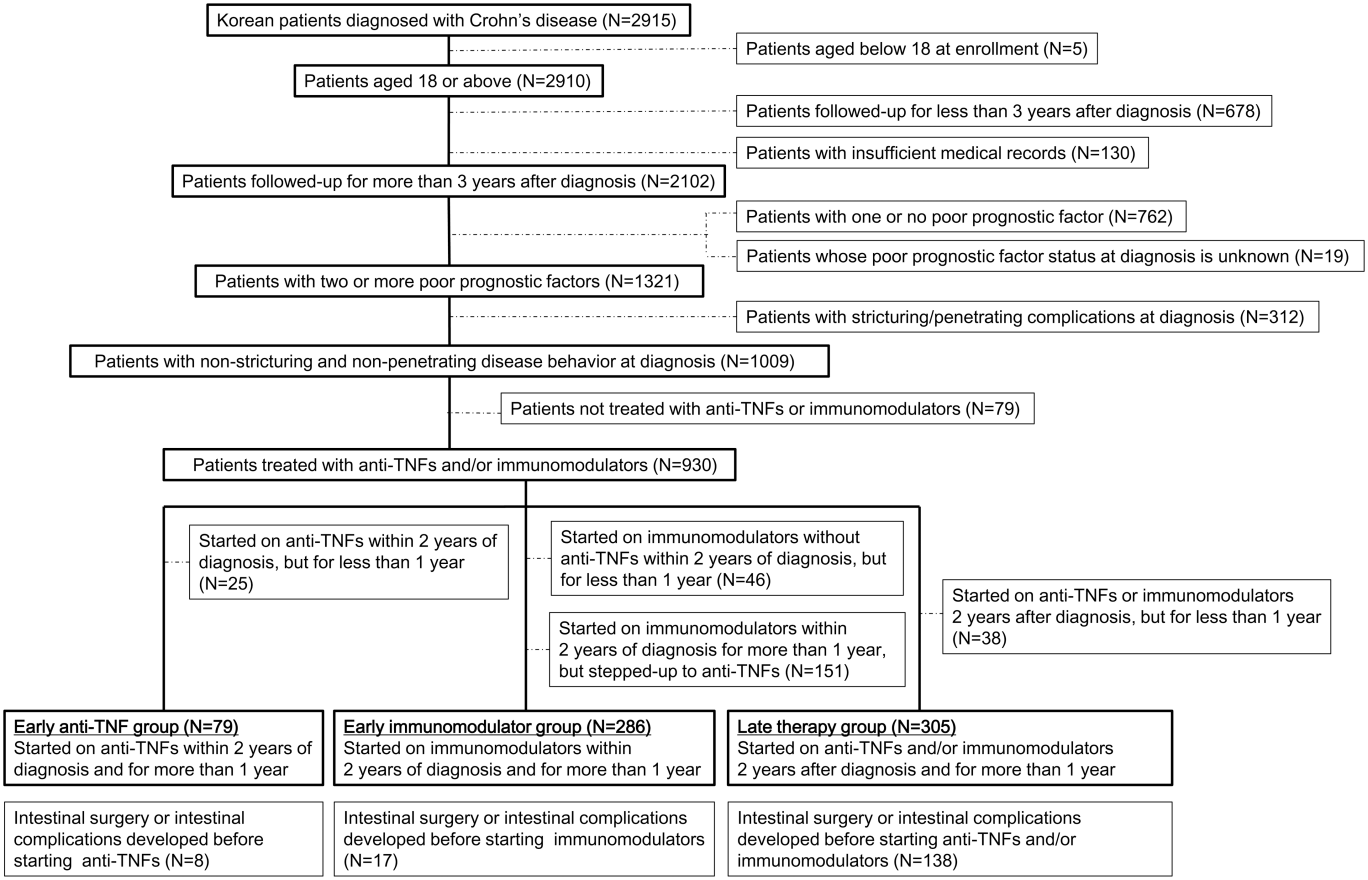
Patient flow diagram.

This retrospective, single-center study involved a review of medical records of patients aged ≥18 years, definitively diagnosed with CD and treated at the IBD center of Asan Medical Center, a tertiary referral hospital in Seoul, Korea, between January 1997 and July 2016. CD was definitively diagnosed based on patients’ clinical features, laboratory findings, endoscopic features, radiologic features, histologic findings, imaging test results, and surgical findings [22]. Patients were included if they had at least two of the following risk factors for progression; diagnosis at age <40 years, need for systemic corticosteroids <3 months after diagnosis, and perianal fistula at diagnosis [19, 20, 23]. Patients followed up for <36 months after diagnosis, and those with insufficient medical records before referrals, were excluded, as were patients who were never treated with an anti-TNF/IM or who had evidence of stricturing and/or penetrating complications before or at diagnosis of CD (Fig 1).

### Data collection

The medical records of all patients were reviewed. Factors recorded included baseline characteristics (age at diagnosis of CD, sex, smoking status at diagnosis, family history of IBD, and follow-up duration after diagnosis), disease characteristics (Montreal disease location without considering upper GI involvement and behavior at diagnosis) [22], and predictors of poor prognosis (need for systemic corticosteroids <3 months after diagnosis, and perianal fistula at diagnosis). Other factors recorded included medications (time to first use of systemic corticosteroids, anti-TNF agents, and IMs) and outcomes (intestinal surgery, behavioral progression, stricturing complications, penetrating complications, development of colorectal cancer, and mortality). The primary outcome variable was intestinal surgery, including intestinal resection and stricturoplasty, after the start of anti-TNF or IM treatment. Secondary outcome variables included behavioral progression, stricturing complications, penetrating complications, colorectal cancer, and mortality, after the start of anti-TNF or IM treatment. Behavioral progression was defined as the development of stricturing or penetrating complications in patients with non-stricturing, non-penetrating behavior (B1) [22].

### Classification of the patients into groups

Patients were classified into three groups according to the timing of anti-TNF/IM therapy. The early anti-TNF group consisted of patients started on anti-TNFs, with or without IMs, within 2 years of CD diagnosis; the early IM group consisted of patients started on IMs without anti-TNFs within 2 years of CD diagnosis; and the late therapy group consisted of patients started on anti-TNFs and/or IMs >2 years after CD diagnosis.

Because the duration and combination of drugs cannot be controlled in a retrospectively designed study, patients were excluded from the study if they remained on anti-TNFs/IMs for <1 year for reasons such as adverse events, costs, reimbursement issues, poor adherence to treatment, or surgery, as the effects of therapy on long-term outcomes could not be assured with short-term treatment [6, 24].

Patients who were started with IM within 2 years of CD diagnosis, but were stepped up to anti-TNF agents were excluded from the early IM group, thereby excluding the influence of anti-TNFs on the course of disease. Combined use of anti-TNF and IM was permitted in the early anti-TNF and late therapy groups (Fig 1).

Patients who were classified into 3 groups, but required intestinal surgery and/or developed intestinal complications (stricturing or penetrating complication) before the start of anti-TNF/IM therapy, were excluded from outcome analyses (Fig 1).

The study protocol was approved by the Institutional Review Board of Asan Medical Center (IRB no. 2016–1119).

### Statistical analysis

Demographic and baseline characteristics were summarized using descriptive statistics. Categorical or nominal variables were described as numbers and percentage and continuous variables as mean ± standard deviation (SD) or median (interquartile range [IQR]), where appropriate. Differences among the three groups were compared using chi-squared tests, linear-by-linear association, or ANOVA (analysis of variance). The Kaplan-Meier method was used to estimate times from starting drug use to the development of outcome events, with groups compared by log rank tests. Cox proportional hazard regression analysis was also performed to evaluate risk factors associated with outcome events. Variables with *P* < 0.1 by univariate analysis were included in multivariate analysis. Statistical significance was defined as a *P* value < 0.05. All statistical analyses were performed using SPSS statistics version 23.0 for Windows (IBM, New York, NY, USA).

## Results

Of the 2,915 patients definitively diagnosed with CD during the study period, 813 were excluded, five because they were younger than 18 years at study enrollment, 678 because they were followed up for <36 months, and 130 because their medical records were incomplete. Of the remaining 2,102 patients, 1,321 had two or more poor prognostic factors. Of the latter, 312 were excluded because they showed stricturing and/or penetrating complications at diagnosis of CD, and 79 were excluded because they had never been treated with anti-TNFs or IMs. The remaining 930 patients were categorized into three groups, the early anti-TNF (n = 104), early IM (n = 483), and late treatment (n = 343) groups. Of these, 25, 197, and 38 patients, respectively, were excluded because they had been treated for <1 year or were stepped up to anti-TNFs. Finally, 670 patients were included, 79 in the early anti-TNF, 286 in the early IM, and 305 in the late therapy group (Fig 1).

### Baseline characteristics of the patient groups

The baseline characteristics of the patients are presented in Table 1. Of the 670 patients, 498 (74.3%) were males. Their mean age at diagnosis of CD was 21.8±6.3 years (Table 1). The early anti-TNF, early IM, and late therapy groups differed significantly in family history of IBD (15.2% vs. 7.0% vs. 5.2%, *P* = 0.009), use of systemic corticosteroids (82.3% vs. 68.9% vs. 77.7%, *P* < 0.001), treatment with IMs (91.1% vs. 100% vs. 95.4%, *P* < 0.001), and treatment with anti-TNF agents (100% vs. 0% vs. 50.5%, *P* < 0.001). Their mean time intervals to first use of systemic corticosteroids (1.74±7.63 months vs. 2.35±8.64 months vs. 19.85±38.52 months, *P* < 0.001), and IMs (5.97±14.35 months vs. 5.02±6.27 months vs. 71.08±44.12 months, *P* < 0.001) differed significantly, as did times to first use of anti-TNFs by patients in the early anti-TNF and late therapy groups (11.56±6.28 months vs. 109.40±58.34 months, *P* < 0.001) (Tables 1 and 2).

**Table 1:** Characteristics of 670 patients with Crohn’s disease and poor prognostic factors.

	Early anti-TNF group (n = 79)	Early IM group (n = 286)	Late therapy group (n = 305)	Total (n = 670)	*P* value
**Male**	57 (72.2%)	232 (81.1%)	209 (68.5%)	498 (74.3%)	**0.002**
**Mean age at diagnosis, years (mean±SD)** **≤16** **≥17–40**	21.0±5.8 20 (25.3%) 59 (74.7%)	22.0±6.5 49 (17.1%) 237 (82.9%)	22.0±6.3 59 (19.3%) 246 (80.7%)	21.8±6.3 128 (19.1%) 542 (80.9%)	0.669 0.259
**Smoking status** **Never smoker** **Ex-smoker** **Current smoker**	59 (74.7%) 5 (6.3%) 15 (19.0%)	202 (70.6%) 17 (6.0%) 67 (23.4%)	217 (71.1%) 14 (4.6%) 74 (24.3%)	478 (71.3%) 36 (5.4%) 156 (23.3%)	0.817
**Family history of IBD**	12 (15.2%)	20 (7.0%)	16 (5.2%)	48 (7.2%)	**0.009**
**Location at diagnosis** **Ileum only (L1)** **Colon only (L2)** **Ileocolon (L3)**	11 (13.9%) 3 (3.8%) 65 (82.3%)	29 (10.1%) 16 (5.6%) 241 (84.3%)	43 (14.1%) 18 (5.9%) 244 (80.0%)	83 (12.4%) 37 (5.5%) 550 (82.1%)	0.579
**Behavior at diagnosis** **Non-stricturing and non-penetrating (B1)** **Stricturing (B2)** **Penetrating (B3)**	79 (100%)	286 (100%)	305 (100%)	670 (100%)	
**Perianal fistula at diagnosis** **Status of perianal fistula at diagnosis** **Healed state** **Active state** **Unknown state**	54 (68.4%) 14 (17.7%) 39 (49.4%) 1 (1.3%)	186 (65.0%) 56 (19.6%) 129 (45.1%) 1 (0.3%)	212 (69.5%) 87 (28.5%) 123 (40.3%) 2 (0.7%)	452 (67.5%) 157 (23.4%) 291 (43.4%) 4 (0.7%)	0.502 0.142 **0.016** 0.265 0.635
**Corticosteroid use** **Time to corticosteroid use, months (mean±SD)**	65 (82.3%) 1.74±7.63	197 (68.9%) 2.35±8.64	237 (77.7%) 19.85±38.52	499 (74.5%) 10.58±28.60	**< 0.001** **< 0.001**
**IM use** **Time to IM use, months (mean±SD)**	72 (91.1%) 5.97±14.35	286 (100%) 5.02±6.27	291 (95.4%) 71.08±44.12	649 (96.9%) 34.75±44.56	**< 0.001** **< 0.001**
**Anti-TNF use** **Time to anti-TNF, months (mean±SD)**	79 (100%) 11.56±6.28	0 (0%) NA	154 (50.5%) 109.40±58.34	233 (34.8%) 76.22±66.43	**< 0.001** **< 0.001**
**Mean follow-up duration, months (mean±SD)**	76.60±31.20	84.72±40.85	157.05±58.29	116.69±61.14	**< 0.001**

IM, immunomodulator; SD, standard deviation; IBD, inflammatory bowel disease; NA, not applicable

**Table 2:** Characteristics of 507 patients with Crohn’s disease and poor prognostic factors, who were not affected by intestinal surgery and/or intestinal complications before starting anti-TNF/IM treatment.

	Early anti-TNF group (n = 71)	Early IM group (n = 269)	Late therapy group (n = 167)	Total (n = 507)	*P* value (among the three groups)	*P* value (early anti-TNF/IM groups vs. late therapy group)
**Male**	52 (73.2%)	217 (80.7%)	117 (70.1%)	386 (76.1%)	**0.034**	**0.003**
**Mean age at diagnosis, yrs, years (mean±SD)** **≤16** **≥17–40**	20.5±5.5 20 (28.2%) 51 (71.8%)	22.0±6.6 48 (17.8%) 221 (82.2%)	21.2±6.4 42 (25.1%) 125 (74.9%)	21.5±6.4 110 (21.7%) 397 (78.3%)	0.072	0.227
**Smoking status** **Never smoker** **Ex-smoker** **Current smoker**	54 (76.1%) 4 (5.6%) 13 (18.3%)	191 (71.0%) 17 (6.3%) 61 (22.7%)	124 (74.3%) 9 (5.4%) 34 (20.4%)	369 (72.8%) 30 (5.9%) 108 (21.3%)	0.904	0.863
**Family history of IBD**	12 (16.9%)	18 (6.7%)	5 (3.0%)	35 (6.9%)	**0.001**	**0.025**
**Location at diagnosis** **Ileum only (L1)** **Colon only (L2)** **Ileocolon (L3)**	9 (12.7%) 3 (4.2%) 59 (83.1%)	26 (9.7%) 14 (5.2%) 229 (85.1%)	22 (13.2%) 11 (6.6%) 134 (80.2%)	57 (11.2%) 28 (5.6%) 422 (83.2%)	0.702	0.448
**Perianal fistula at diagnosis**	49 (69.0%)	173 (64.3%)	114 (68.3%)	336 (66.3%)	0.607	0.572
**Corticosteroid use**	53 (74.6%)	160 (59.5%)	84 (50.3%)	297 (58.6%)	**0.002**	**0.011**
**Mean follow-up duration from diagnosis of CD, months (mean±SD)**	75.21±31.52	84.61±41.07	145.44±55.06	103.33±53.92	**< 0.001**	**< 0.001**

CD, Crohn’s disease; IM, immunomodulator; IBD, inflammatory bowel disease

### Comparisons of outcome variables among the groups

Of the patients in the early anti-TNF, the early IM, and the late therapy groups, 8, 17, and 138 patients, respectively, were excluded from outcome analyses because of intestinal surgery or intestinal complications before starting anti-TNF/IM therapy, which indicates that the disease had already progressed to an advanced or complicated form. Of the remaining 507 patients (Table 2), 83 underwent intestinal surgeries (12 in the early anti-TNF group, 26 in the early IM group, and 45 in the late therapy group), including 82 who underwent intestinal resection and one who underwent small bowel stricturoplasty only. Behavioral progression, stricturing complication, and penetrating complication developed in each group as follows; 13, 10, and 8 in the early anti-TNF group, 66, 57, and 22 in the early IM group, and 73, 56, and 47 in the late therapy group. After starting anti-TNF/IM therapy, two patients, all in the late therapy group, were diagnosed with colorectal cancer, and four patients died during follow-up, including two who died of causes unrelated to CD; one in the early IM group (brain tumor), and one in the late therapy group (traffic accident). Two patients in the late therapy group died of CD-related causes; one of septic shock and one of rectal cancer.

### Time to the outcomes by the groups

**Fig 2:**
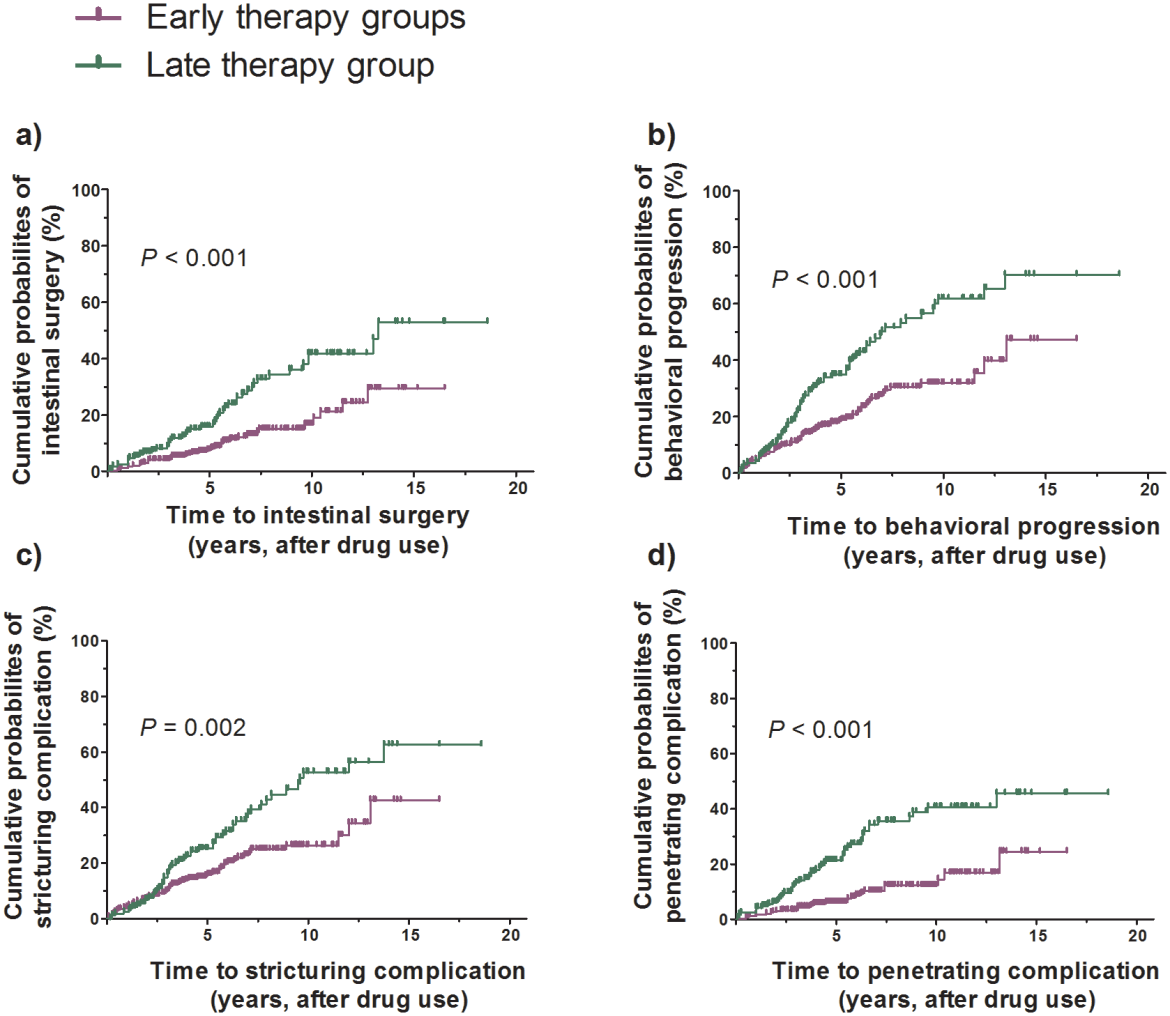
The Kaplan-Meier survival analysis for key outcome variables from starting anti-TNFs/IMs treatment. (A) Cumulative probabilities of intestinal surgery (the early anti-TNF/IM groups vs. the late therapy group, *P* < 0.001), (B) Cumulative probabilities of behavioral progression (the early anti-TNF/IM groups vs. the late therapy group, *P* < 0.001), (C) Cumulative probabilities of developing stricturing complication (the early anti-TNF/IM groups vs. the late therapy group, *P* = 0.002), and (D) Cumulative probabilities of developing penetrating complication (the early anti-TNF/IM groups vs. the late therapy group, *P* < 0.001) from starting anti-TNFs/IMs.

Kaplan-Meier analysis showed that, from starting anti-TNFs/IMs treatment, times to development of all outcome events differed significantly among the three groups, including cumulative rates of intestinal surgery (*P <* 0.001), behavioral progression (*P <* 0.001), stricturing complications (*P* = 0.008), and penetrating complications (*P <* 0.001). Significant differences were also observed when the early anti-TNF/IM groups were compared with the late therapy group in terms of cumulative rates of intestinal surgery (*P <* 0.001), behavioral progression (*P <* 0.001), stricturing complications (*P* = 0.002), and penetrating complications (*P <* 0.001) from starting anti-TNFs/IMs. Although the cumulative probabilities of development of all outcomes were significantly higher in the late therapy group than in the early anti-TNF and/or early IM groups, there were no consistent differences between the early anti-TNF and early IM groups (Fig 2a, 2b, 2c and 2d).

### Hazard ratios (HRs) for variables affecting outcomes

Univariate analysis showed that male sex was significantly associated with behavioral progression (hazard ratio [HR]: 0.695, 95% confidence interval [CI]: 0.488–0.991, *P* = 0.044) and stricturing complications (HR: 0.641, 95% CI: 0.436–0.943, *P* = 0.024). Colonic disease (L2) was also associated with behavioral progression (HR: 0.241, 95% CI: 0.083–0.701, *P* = 0.009) and stricturing complications (HR: 0.248, 95% CI: 0.073–0.849, *P* = 0.026). In addition, late therapy was significantly associated with intestinal surgery (HR: 2.318, 95% CI: 1.503–3.577, *P <* 0.001), behavioral progression (HR: 2.044, 95% CI: 1.486–2.813, *P <* 0.001), stricturing complications (HR: 1.732, 95% CI: 1.213–2.473, *P* = 0.003), and penetrating complications (HR: 3.315, 95% CI: 2.094–5.249, *P <* 0.001), from starting anti-TNFs/IMs (Tables 3–6).

**Table 3:** Cox regression analysis showing hazard ratios (HRs) for variables affecting intestinal surgery.

	n (%)	Univariate analysis			Multivariate analysis					
		aHR	95% CI	*P* value	aHR	95% CI	*P* value	aHR	95% CI	*P* value
Male sex	386/507 (76.1%)	0.783	0.484–1.267	0.320	Not retained			Not retained		
Age at diagnosis, years ≤16 ≥17–40	110/507 (21.7%) 397/507 (78.3%)	reference 0.727	0.446–1.184	0.200	Not retained			Not retained		
Smoking status Never smoker Ex-smoker Current smoker	369/507 (72.8%) 30/507 (5.9%) 108/507 (21.3%)	reference 1.964 1.041	0.970–3.979 0.617–1.757	**0.061** 0.880	reference 1.964 1.117	0.970–3.979 0.662–1.886	0.061 0.679	reference 1.963 1.100	0.969–3.977 0.651–1.856	0.061 0.722
Family history of IBD	35/507 (6.9%)	0.565	0.178–1.791	0.332	Not retained			Not retained		
Location at diagnosis Ileum only (L1) Colon only (L2) Ileocolon (L3)	57/507 (11.2%) 28/507 (5.5%) 422/507 (83.2%)	reference 0.509 0.674	0.166–1.564 0.372–1.223	0.239 0.194	Not retained			Not retained		
Perianal fistula at diagnosis	336/507 (66.3%)	1.233	0.775–1.962	0.378	Not retained			Not retained		
Corticosteroid use <3 months after diagnosis	297/507 (58.6%)	1.137	0.728–1.776	0.572	Not retained			Not retained		
Group Early anti-TNF group Early IM group Late therapy group Late therapy group relative to the early anti-TNF/IM groups	71/507 (14.0%) 269/507 (53.1%) 167/507 (32.9%)	reference 0.406 1.153 2.318	0.204–0.807 0.605–2.198 1.503–3.577	**0.010** 0.665 **< 0.001**	reference 0.405 1.154	0.204–0.806 0.606–2.199	**0.010** 0.663	2.321	1.503–3.584	**< 0.001**

HR, hazard ratio; CI, confidence interval; IM, immunomodulator; IBD, inflammatory bowel disease.

**Table 4:** Cox regression analysis showing hazard ratios (HRs) for variables affecting behavioral progression.

	n (%)	Univariate analysis			Multivariate analysis					
		aHR	95% CI	*P* value	aHR	95% CI	*P* value	aHR	95% CI	*P* value
Male sex	386/507 (76.1%)	0.695	0.488–0.991	**0.044**	0.722	0.503–1.034	0.076	0.728	0.508–1.042	0.083
Age at diagnosis, years ≤16 ≥17–40	110/507 (21.7%) 397/507 (78.3%)	reference 1.047	0.709–1.548	0.817	Not retained			Not retained		
Smoking status Never smoker Ex-smoker Current smoker	369/507 (72.8%) 30/507 (5.9%) 108/507 (21.3%)	reference 0.837 1.052	0.408–1.718 0.721–1.536	0.628 0.791	Not retained			Not retained		
Family history of IBD	35/507 (6.9%)	0.887	0.452–1.740	0.727	Not retained			Not retained		
Location at diagnosis Ileum only (L1) Colon only (L2) Ileocolon (L3)	57/507 (11.2%) 28/507 (5.5%) 422/507 (83.2%)	reference 0.241 0.652	0.083–0.701 0.414–1.025	**0.009** **0.064**	reference 0.213 0.672	0.073–0.621 0.426–1.059	**0.005** 0.087	reference 0.213 0.672	0.073–0.623 0.426–1.060	**0.005** 0.088
Perianal fistula at diagnosis	336/507 (66.3%)	0.949	0.680–1.325	0.759	Not retained			Not retained		
Corticosteroid use <3 months after diagnosis	297/507 (58.6%)	1.146	0.825–1.590	0.416	Not retained			Not retained		
Group Early anti-TNF group Early IM group Late therapy group Late therapy group relative to the early anti-TNF/IM groups	71/507 (14.0%) 269/507 (53.1%) 167/507 (32.9%)	reference 1.134 2.269 2.044	0.625–2.058 1.256–4.101 1.486–2.813	0.678 **0.007** **< 0.001**	reference 1.191 2.309	0.655–2.166 1.277–4.174	0.566 **0.006**	2.001	1.449–2.763	**< 0.001**

HR, hazard ratio; CI, confidence interval; IM, immunomodulator; IBD, inflammatory bowel disease.

**Table 5:** Cox regression analysis showing hazard ratios (HRs) for variables affecting stricturing complications.

	n (%)	Univariate analysis			Multivariate analysis					
		aHR	95% CI	*P* value	aHR	95% CI	*P* value	aHR	95% CI	*P* value
Male sex	386/507 (76.1%)	0.641	0.436–0.943	**0.024**	0.649	0.438–0.961	**0.031**	0.655	0.443–0.969	**0.034**
Age at diagnosis, years ≤16 ≥17–40	110/507 (21.7%) 397/507 (78.3%)	reference 1.501	0.929–2.426	**0.097**	reference 1.583	0.976–2.566	0.063	reference 1.598	0.986–2.589	0.057
Smoking status Never smoker Ex-smoker Current smoker	369/507 (72.8%) 30/507 (5.9%) 108/507 (21.3%)	reference 0.908 1.035	0.420–1.963 0.679–1.577	0.806 0.874	Not retained			Not retained		
Family history of IBD	35/507 (6.9%)	0.993	0.485–2.034	0.984	Not retained			Not retained		
Location at diagnosis Ileum only (L1) Colon only (L2) Ileocolon (L3)	57/507 (11.2%) 28/507 (5.5%) 422/507 (83.2%)	reference 0.248 0.707	0.073–0.849 0.423–1.181	**0.026** 0.185	reference 0.212 0.728	0.062–0.729 0.434–1.220	**0.014** 0.228	reference 0.212 0.730	0.062–0.731 0.436–1.224	**0.014** 0.232
Perianal fistula at diagnosis	336/507 (66.3%)	0.843	0.585–1.215	0.360	Not retained			Not retained		
Corticosteroid use <3 months after diagnosis	297/507 (58.6%)	1.311	0.905–1.901	0.152	Not retained			Not retained		
Group Early anti-TNF group Early IM group Late therapy group Late therapy group relative to the early anti-TNF/IM groups	71/507 (14.0%) 269/507 (53.1%) 167/507 (32.9%)	reference 1.260 2.102 1.732	0.643–2.471 1.070–4.131 1.213–2.473	0.501 **0.031** **0.003**	reference 1.288 2.141	0.656–2.531 1.089–4.210	0.462 **0.027**	1.736	1.209–2.493	**0.003**

HR, hazard ratio; CI, confidence interval; IM, immunomodulator; IBD, inflammatory bowel disease.

**Table 6:** Cox regression analysis showing hazard ratios (HRs) for variables affecting penetrating complications.

	n (%)	Univariate analysis		
		aHR	95% CI	*P* value
Male sex	386/507 (76.1%)	0.685	0.420–1.117	0.130
Age at diagnosis, years ≤16 ≥17–40	110/507 (21.7%) 397/507 (78.3%)	reference 0.725	0.439–1.197	0.209
Smoking status Never smoker Ex-smoker Current smoker	369/507 (72.8%) 30/507 (5.9%) 108/507 (21.3%)	reference 1.740 1.166	0.789–3.837 0.688–1.976	0.170 0.568
Family history of IBD	35/507 (6.9%)	0.384	0.094–1.565	0.182
Location at diagnosis Ileum only (L1) Colon only (L2) Ileocolon (L3)	57/507 (11.2%) 28/507 (5.5%) 422/507 (83.2%)	reference 0.442 0.698	0.123–1.586 0.368–1.324	0.211 0.271
Perianal fistula at diagnosis	336/507 (66.3%)	1.006	0.629–1.607	0.981
Corticosteroid use <3 months after diagnosis	297/507 (58.6%)	1.086	0.687–1.718	0.724
Group Early anti-TNF group Early IM group Late therapy group Late therapy group relative to the early anti-TNF/IM groups	71/507 (14.0%) 269/507 (53.1%) 167/507 (32.9%)	reference 0.575 2.147 3.315	0.255–1.294 1.010–4.562 2.094–5.249	0.181 **0.047** **< 0.001**

HR, hazard ratio; CI, confidence interval; IM, immunomodulator; IBD, inflammatory bowel disease.

Multivariate Cox regression analysis showed that, from starting anti-TNFs/IMs, late therapy was independently associated with intestinal surgery (adjusted HR [aHR]: 2.321; 95% CI: 1.503–3.584; *P <* 0.001), behavioral progression (aHR: 2.001; 95% CI: 1.449–2.763; *P <* 0.001), and stricturing complications (aHR: 1.736; 95% CI: 1.209–2.493; *P* = 0.003). Colonic disease (L2) was negatively associated with behavioral progression (aHR: 0.213; 95% CI: 0.073–0.623; *P* = 0.005) and stricturing complications (aHR: 0.212; 95% CI: 0.062–0.731; *P* = 0.014), and male sex was also negatively associated with stricturing complications (aHR: 0.655; 95% CI: 0.443–0.969; *P* = 0.034) (Tables 3–6).

## Discussion

The purpose of this study was to verify the real-life effectiveness of early therapy with anti-TNFs or IMs on improving long-term outcomes in patients with CD and poor prognostic factors, who were naïve to both intestinal surgery and intestinal complications. Starting from anti-TNFs/IMs treatment, the development of all outcome events differed significantly among the three groups of patients, with early anti-TNF/IM treatment consistently associated with better long-term outcomes, as shown by delays in intestinal surgery and intestinal complications. Multivariate analysis also showed that late anti-TNF/IM therapy was independently associated with intestinal surgery and intestinal complications, from starting anti-TNFs/IMs. We also found that colonic involvement was negatively associated with behavioral progression and stricturing complications, revalidating the associations of ileal involvement with behavioral progression and poor outcomes [23, 25].

Anti-TNFs are effective in inducing clinical remission in CD patients with moderate to severe disease activity [14] and in maintaining clinical remission [6, 8]. Before the introduction of anti-TNFs, patients with CD usually received symptom-based therapy, which did not change the long-term course of CD, eventually leading to stricturing and/or penetrating complications [26]. However, as anti-TNFs have been clearly shown to control CD activity, treatment paradigms have changed, with emphasis placed on preventing structural damage and improving disease course by inducing mucosal healing.

In the SONIC trial, combination therapy with infliximab and azathioprine was shown superior to infliximab or azathioprine monotherapy in achieving corticosteroid-free clinical remission and mucosal healing more frequently, while not significantly increasing the risks of infectious complications [27]. In the top-down versus step-up study, induction therapy with combined immunosuppression in patients naïve to corticosteroids resulted in higher probabilities of remission without systemic corticosteroid therapy or surgery at 26 and 52 weeks than conventional management [28]. Moreover, the mucosal healing rate through 2 years was higher in the early combined immunosuppression than in the conventional management group [28]. Recently, the REACT study also found that rates of intestinal surgery, hospital admission, and serious disease-related complications were significantly lower at 24 weeks in patients given early combined immunotherapy, while not increasing serious drug-related adverse events, compared with patients given conventional therapy [29]. These results, showing the efficacy of early anti-TNF therapy, especially combined with IMs, in improving the prognosis of patients with CD, led to the concept of top-down or early intensive therapy in these patients [18, 30].

Recent randomized controlled trials from France and Spain reported that early IM therapy was not superior to conventional therapy in patients with CD [13, 31]. By contrast, real-life cohort studies from Hungary and South Korea showed that early IM therapy significantly reduced the probabilities of intestinal surgery and complications [32, 33].

Despite their clinical effectiveness, anti-TNFs and IMs are associated with troublesome adverse effects, including infection, malignancy, hepatotoxicity, and allergic reactions [15, 16]. As treatment strategies must be individualized, depending on each patient’s comorbidities and prognosis [20], medications should be chosen based on a balance between their efficacy and safety [34]. Selected patients may require early intensive therapy to prevent disease progression and poor outcomes, whereas others may live with well-controlled disease activity without early intensive therapy [35, 36].

Young age at diagnosis, perianal fistula at diagnosis, early need for systemic corticosteroids, ileal involvement, and smoking have been identified as factors predicting poor outcomes of CD [17, 37]. These factors can be used to select patients likely to benefit from more aggressive therapy [38]. The present study used the first three factors as inclusion criteria, because many studies have shown these to be associated with poor prognosis and information about their presence or absence was easy to obtain [39–42].

This study had several strengths. To our knowledge, it is the largest real-life study to date of CD patients from Asian countries. Although several studies have shown the effectiveness of early anti-TNF/IM therapy in Western patients, disease characteristics and medical environments differ markedly between Western and Asian patients. Therefore, results obtained in Western populations may be inapplicable to Asian patients. Furthermore, most previous studies were randomized controlled trials, which have limitations in study duration and may not reflect real-life situations [24, 27–29]. The present study included a large number of patients and long follow-up durations in real clinical situations, enabling us to compare long-term outcomes among groups of patients that differed in the timing of starting anti-TNFs/IMs.

A second strength of this study was its inclusion of patients at a single-center study, standardizing treatment strategies among study subjects. Clinical data were also obtained from a well-established IBD patient registry of our center [33].

Third, the study subjects were limited to those having poor prognostic factors, enabling the effectiveness of early anti-TNF/IM therapy to be analyzed only in these patients. As early intensive therapy should be administered only to patients in whom the benefits outweigh the disadvantages, these results may provide information about the effectiveness of early intensive therapy in patients who really need it [17, 37, 38].

Fourth, the study subjects were limited to those having non-stricturing and non-penetrating behavior (B1) and who were also naïve to intestinal surgery. Most studies assessing the effectiveness of anti-TNFs have enrolled patients with stricturing (B2) or penetrating (B3) complications as well as those without these complications [6, 8, 18]. Patients already affected by intestinal complications or who have undergone intestinal surgery are expected to show poorer responses to potent drugs because structural damage has already developed. By limiting the study patients to those having inflammatory behavior without having undergone previous intestinal surgery, we were able to verify the superiority of early anti-TNF/IM therapy in groups of patients without overt complications. Despite the lack of overt complications, however, insidious bowel damage and/or some irreversible pathophysiologic change may have already progressed in the late therapy group, thereby reducing the efficacy of anti-TNF/IM therapy in these patients. Therefore, this study showed that early anti-TNF/IM therapy should be initiated in patients with poor prognostic factors, despite the absence of overt complications.

The major potential limitation of this study was the possible biased classification of the patients into the three groups, as patients with higher disease activity during the early course of disease may have been classified into the early anti-TNF/IM group rather than the late therapy group. Conventional step-up therapy is being adopted to treat IBD patients in Korea, and anti-TNFs are indicated and reimbursed by the government for use in patients with moderate to severe disease activity after failing conventional medications [20, 43]. Compared with patients in the late therapy group, those in the early anti-TNF/IM groups may have required more intensive treatment after failure of conventional medications during the early course of disease. That is, the disease course in patients in the late therapy group may have been relatively quiescent, with less need for more intensive therapy. Because their early disease activity was higher, patients in the early anti-TNF/IM groups may have been expected to have poorer outcomes. This explanation is supported by the differences in mean time intervals to first use of systemic corticosteroids among the groups which is presented in Table 1. Nevertheless, our results showed that all poor outcome events were less common in the early anti-TNF/IM groups than in the late therapy group. This finding indicates that the expected poorer long-term outcomes in the early INF/IM groups were overcome by early anti-TNF/IM therapy, despite their higher early disease activity. An idea of adjusting disease activity among groups using indices such as Crohn’s disease activity index (CDAI) or serum C-reactive protein level could be suggested to overcome our limitation. However, it was not possible because the information at the time of diagnosis or starting treatment could not be obtained in all patients as most of study subjects were referred from other hospitals. Therefore, if severity of disease and baseline characteristics should be controlled among groups for more accurate comparisons, prospective randomized controlled trials would be ideal, even though it has limitations in evaluating long-term outcomes.

A second limitation was that we did not show a difference between early use of anti-TNFs and IMs. Because anti-TNFs are more potent than IMs in controlling disease activity in IBD patients [12, 14], we expected that early use of anti-TNFs would yield better outcomes than early use of IMs only. However, patients with more aggressive early disease may have been classified into the early anti-TNF rather than the early IM group for the reasons cited in the previous paragraph. Furthermore, as “anti-TNF top-down therapy” is not reimbursed in Korea, no patient in this study received “anti-TNF top-down therapy”. The patients in the early anti-TNF group were given anti-TNFs after failure of or intolerance to IMs rather than being treated initially with anti-TNFs. Although the patients in the early anti-TNF group had higher early disease activity not controlled by IMs, the outcomes were not consistently poorer in the early anti-TNF than in the early IM group. These findings suggest that application of “anti-TNF top-down therapy” to the early anti-TNF group, rather than anti-TNF treatment after failure of or intolerance to IMs, would have yielded better outcomes than early IMs alone. Large-scale, long-term randomized controlled trials comparing anti-TNF top-down, accelerated step-up, and classical step-up strategies in CD patients with poor prognostic factors are therefore needed [28, 29, 44].

A third limitation was the retrospective design of this study, despite the prospective enrollment of patients in our IBD patient registry. However, actual data were maintained by IBD specialists, who updated clinical information when specific events occurred, thereby increasing the reliability of our data. Because this study was retrospective in nature, some information depended on patient recall, especially regarding events before referral to our hospital.

A fourth limitation may have been referral bias, because all included patients were enrolled from a single, tertiary referral hospital. However, because most CD patients in South Korea are continuously and regularly followed at referral centers, not referred back to primary or secondary medical institutions, the patients in this study may represent the general CD patient population of South Korea.

Finally, patient assignment to groups differed by years of CD diagnosis. Of the 91 patients diagnosed with CD from 1985 to 1999, 86 (94.5%) were enrolled in the late therapy group and none in the early anti-TNF group. By contrast, of the 391 patients diagnosed from 2000 to 2009, 36 (9.0%) were enrolled in the early anti-TNF group and 149 (38.1%) in the early IM group; and of the 188 patients diagnosed after 2010, 176 (93.6%) were assigned to the early anti-TNF/IM groups. Differences in patient proportions among the three groups according to year of diagnosis may reflect the unavailability of anti-TNFs before 2000. Furthermore, studies showing the effectiveness of anti-TNFs/IMs have altered CD treatment paradigms, which likely affected patient assignments.

In conclusion, starting from anti-TNF or IM treatment, CD patients with poor prognostic factors who receive early treatment would have a longer time to experience their first intestinal surgery and first intestinal complications compared to those who receive late treatment with anti-TNFs or IMs. Further large-scale, prospective, long-term, randomized controlled trials in Asian countries are warranted to assess the efficacy of early anti-TNF therapy.
